# GaAs Cone-Shell Quantum Dots in a Lateral Electric Field: Exciton Stark-Shift, Lifetime, and Fine-Structure Splitting

**DOI:** 10.3390/nano14141174

**Published:** 2024-07-10

**Authors:** Ahmed Alshaikh, Robert H. Blick, Christian Heyn

**Affiliations:** Center for Hybrid Nanostructures (CHyN), University of Hamburg, Luruper Chaussee 149, 22761 Hamburg, Germany; ahmed.alshaikh@uni-hamburg.de (A.A.); rblick@physnet.uni-hamburg.de (R.H.B.)

**Keywords:** quantum dot, photoluminescence, exciton, lateral electric field, exciton energy, Stark-shift, exciton lifetime, exciton fine-structure splitting

## Abstract

Strain-free GaAs cone-shell quantum dots have a unique shape, which allows a wide tunability of the charge-carrier probability densities by external electric and magnetic fields. Here, the influence of a lateral electric field on the optical emission is studied experimentally using simulations. The simulations predict that the electron and hole form a lateral dipole when subjected to a lateral electric field. To evaluate this prediction experimentally, we integrate the dots in a lateral gate geometry and measure the Stark-shift of the exciton energy, the exciton intensity, the radiative lifetime, and the fine-structure splitting (FSS) using single-dot photoluminescence spectroscopy. The respective gate voltage dependencies show nontrivial trends with three pronounced regimes. We assume that the respective dominant processes are charge-carrier deformation at a low gate voltage *U*, a vertical charge-carrier shift at medium *U*, and a lateral charge-carrier polarization at high *U*. The lateral polarization forms a dipole, which can either enhance or compensate the intrinsic FSS induced by the QD shape anisotropy, dependent on the in-plane orientation of the electric field. Furthermore, the data show that the biexciton peak can be suppressed by a lateral gate voltage, and we assume the presence of an additional vertical electric field induced by surface charges.

## 1. Introduction

Epitaxially grown semiconductor quantum dots (QDs) are highly attractive for fundamental research as well as for advanced applications [1]. With a size smaller than the De Broglie wavelength of the embedded charge carriers, quantization effects become significant yielding, e.g., a discretization of the optical emission lines. The energy of the optical emission can be tailored by the fabrication process via the QD size, shape, and composition [2]. Even after the fabrication, the quantized energy states and, thus, the emission of the QDs can be tuned by strain [3], external electric [4,5,6,7,8,9], or magnetic [10,11,12,13,14] fields, and by the temperature [15,16,17,18].

This wide control on the quantized energy states and their optical stability suggests QDs as essential building blocks for applications in quantum information technology, where they intrigue with their on-demand emission of single photons [19,20]. For instance, quantum cryptography using the BB84 protocol [21] is based on entangled photons, where recently, the highly efficient extraction of entangled photons from QDs has been demonstrated [22]. For an entanglement, the photons must be indistinguishable, which requires a negligible exciton fine-structure splitting (FSS) [23,24,25,26]. The FSS is caused by anisotropies, e.g., of the QD shape [26], of strain-induced piezoelectric fields [27,28], and of the atomistic crystal symmetry [29,30]. The resulting anisotropic in-plane electron-hole exchange interaction lifts the degeneracy of the bright exciton pairs and splits them into two orthogonally polarized components [31,32]. Several methods for a minimization of the FSS have been studied, like a QD shape selection [26], external strain [22], or an in-plane (lateral) electric field [33,34,35]. We note that here rather large QDs with a large FSS [26] are studied. However, as an important advantage, the large size allows a strong charge-carrier tunability. The goal is not a minimization of the FSS, but instead their usage as a sensor for the lateral anisotropy.

GaAs cone-shell quantum dots (CSQDs) are in many aspects different from InAs QDs, where the influence of a lateral electric field was already studied [34,35]. As a first point, the GaAs CSQDs are almost strain-free and, thus, a prominent source for an anisotropy can be neglected. Furthermore, the CSQDs have a unique shape that allows a wide charge-carrier tunability by external electric or magnetic fields. In a vertical electric field, the transformation from a QD into a quantum ring is predicted [9] and combined with an additional magnetic field the usage as a switchable trap for photoexcited charge carriers [14]. For the present lateral electric field, simulations predict the transformation of a localized exciton into a lateral dipole. In order to evaluate this prediction experimentally, we have integrated CSQDs into a lateral gate structure and measured the influence of the gate voltage on the exciton energy and intensity, on the radiative lifetime, and on the FSS.

## 2. Methods

The studied cone-shell quantum dots are fabricated in a self-assembled fashion using local droplet etching (LDE) during molecular beam epitaxy (MBE) [9,36]. In brief, Al droplets are deposited on an Al_0.34_Ga_0.66_As surface and drill low-density (about $1\times 10^{7}$ cm^−2^) nanoholes into the surface during a post-growth annealing step. Subsequently, the nanoholes are filled by deposition of a GaAs layer for CSQD generation. That means the bottom part of a CSQD is formed by the nanohole template and the top part by capillarity. The thickness $d_{F}$ of the GaAs filling layer controls the CSQD height, which is in very rough approximation $15d_{F}$ [37]. The present dots are formed with $d_{F}$ = 0.64 nm. Finally, the layer with the CSQDs is capped with 80 nm Al_0.34_Ga_0.66_As.

**Figure 1:**
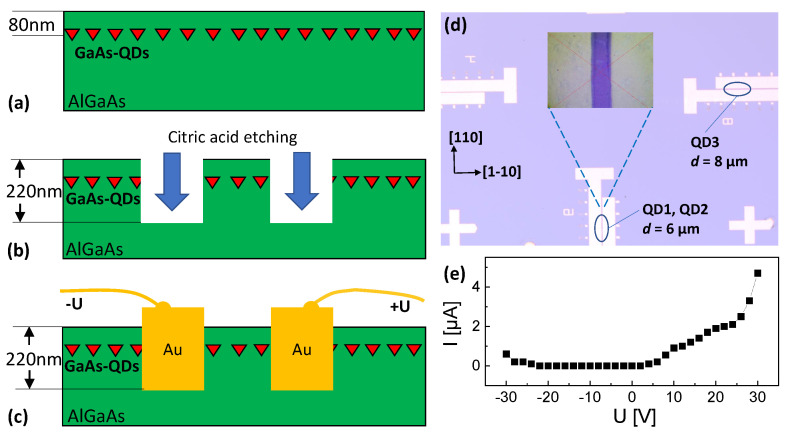
Cross-sectional schematics of the fabrication steps for the lateral gates. (**a**) MBE grown layer sequence with an embedded layer containing the GaAs cone-shell QDs. (**b**) Citric acid etching of the trenches for the gates. (**c**) Filling of the trenches with Au. (**d**) Top view schematics and microscopy image (insert) of the lateral gate-structure. The crystalline directions, gate distances *d*, and the positions of the studied QDs are indicated. (**e**) Measured current-voltage characteristics of the gate structure with *d* = 6 μm.

For the lateral gate geometry (Figure 1), trenches are etched with citric acid into the MBE-grown samples using optical lithography. Afterwards, the trenches are filled with Au. Two gate structures with respective gate distances of *d* = 6 µm and *d* = 8 µm are used for the present measurements. The *d* = 6 µm gate structure has an orientation of the electric field along the [1-10]-direction and the *d* = 8 µm gate structure along the [110]-direction. The inset in Figure 1d shows a top-view microscopy image of a gate. Visible is a slight non-uniformity which can cause a local variation of *d* and, thus, of the induced electric field *F*. In Figure 1, a current-voltage measurement between two gate electrodes is plotted, which establishes the only negligible leakage current in the studied voltage range up to ±30 V.

Single-dot microphotoluminescence (PL) is used for the characterization of the optical emission from the CSQDs. The samples are installed in an optical closed-cycle cryostat (Montana Cryostation S100, Bozeman, MT, USA) at a temperature of *T* = 5 K. A stack of piezo motors is integrated inside the cryostat for sample movement. The exciting laser has an energy of 2.43 eV and can be used in continuous wave (CW) or a pulsed mode, with 100 ps pulse length and 10 ns repetition time. For focusing the laser and collecting the light emitted from the CSQDs, an objective LMPLFLN-BD, 100 × 0.8 (Olympus, Tokio, Japan) is installed inside the cryostat. The low QD density allows the selection of individual CSQDs without aperture by the focused laser. Two spectrometers are used for the measurements. A *f* = 750 mm monochromator Acton SP-2750 (Princeton Instruments, Acton, MA, USA) with a cooled camera for spectral analysis and a *f* = 500 mm monochromator Shamrock SR 500i (Andor—Oxford Instruments, Abingdon, UK) equipped with an avalanche photodiode (APD) for the lifetime measurements are used. For polarization-dependent measurements, the emitted light is analyzed by a rotatable λ/2 waveplate followed by a fixed polarizer.

The finite-element simulation of the exciton ground-state energy in a CSQD is based on effective mass approximation and expands an earlier model which is described in Ref. [9]. Enhancements in the present model are a full three-dimensional meshing and solver, realized by a self-written Python code. Furthermore, correlation effects are now considered in a self-consistent fashion. A cubic meshing in Cartesian coordinates discretizes the potential with a resolution of 0.8 nm. The potential of an individual mesh cell at a position vector *r* is $V_{e}\left(r\right)=V_{0}+V_{F,e}-V_{C,h}$ for electrons and $V_{h}\left(r\right)=V_{0}+V_{F,h}-V_{C,e}$ for holes, where $V_{0}$ is zero inside the GaAs CSQD, and inside the confining AlGaAs barrier $V_{0}$ = 0.286 eV for electrons and $V_{0}$ = 0.168 eV for holes. The potentials induced by the lateral electric field *F* in *x*-direction are $V_{F,e}=qxF$ and $V_{F,h}=-qxF$, where *q* is the elementary charge. $V_{C,e}$ and $V_{C,h}$ consider correlation effects via the potential induced by the additional charge carrier inside the dot. A simulation run starts with $V_{C,e}=V_{C,h}=0$ and computes the electron and hole single-particle wave functions $\Psi_{e}$, $\Psi_{h}$ by solving the Schrödinger equation in matrix notation using the finite-difference method [38]. The electron-effective masses are $m_{e}^{*}=0.067m_{0}$ inside the GaAs CSQS and $m_{e}^{*}=0.086m_{0}$ inside the AlGaAs barrier, with the free electron mass $m_{0}$. The hole masses are $m_{h}^{*}=0.51m_{0}$ and $m_{h}^{*}=0.59m_{0}$, respectively. From the computed single-particle wave functions, the electron and hole charge distributions $p_{e}\left(r\right)=q{\left|\Psi_{e}\left(r\right)\right|}^{2}$ and $p_{h}\left(r\right)=q{\left|\Psi_{h}\left(r\right)\right|}^{2}$ are calculated. This gives the potentials $V_{C,e}\left(r\right)$ and $V_{C,h}\left(r\right)$ via Poisson’s equation. In the next simulation step, the Schrödinger equation is solved again to determine the electron and hole wave functions and the corresponding eigenenergies $E_{e}$, $E_{h}$, but now under consideration of the potential induced by the additional charge carrier. In detail, the electron is computed considering the additional hole via $V_{C,h}$ and vice versa. From the simulated wave functions, we calculate the Coulomb interaction energy $C_{eh}$ between the electron and the hole using the Coulomb integral. Finally, the exciton recombination energy is taken from $E_{X}=E_{g}+E_{e}+E_{h}-C_{eh}$, with the GaAs band-gap energy $E_{g}$.

## 3. Simulation Results

**Figure 2:**
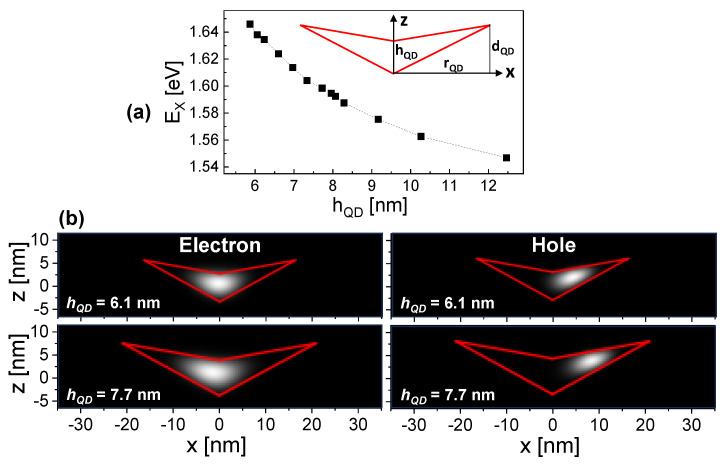
(**a**) Simulated exciton energy $E_{X}$ as function of the QD height $h_{QD}$ at zero *F*. The inset shows a cross-sectional sketch of the rotational-symmetric CSQD shape. (**b**) Simulated cross-sectional electron and hole probability densities for CSQDs with different height $h_{QD}$ in a lateral electric field *F* = 30 kV/cm. The red lines indicate the assumed cross-sectional QD shape with (**top**): $h_{QD}$ = 6.1 nm, $d_{QD}$ = 9.0 nm, $r_{QD}$ = 16.5 nm, and (**bottom**): $h_{QD}$ = 7.7 nm, $d_{QD}$ = 11.4 nm, $r_{QD}$ = 21.1 nm.

We start the results part with the outcome of the simulation since the predicted formation of a lateral dipole represents a major motivation for this study. For the simulations, the CSQD shape is assumed according to Ref. [9], where CSQDs are studied using a combination of atomic force microscopy (AFM), PL measurements in a vertical electric field, and simulations. There, the shape is approximated as a cone with a cone-shaped indentation on the top. Assuming a cone-shaped indentation like in Ref. [9], the CSQD size is characterized by only one fitting parameter which is the height $h_{QD}$ at the CSQD center (inset of Figure 2a).

Figure 2a shows the simulated relation between the CSQD height $h_{QD}$ and the corresponding exciton energy $E_{X}$ at zero field. These data illustrate that the CSQD emissions can be tailored over a wide range by the nanohole-filling level $d_{F}$ [37]. For the CSQDs discussed in Section 4, exciton energies are measured that agree with $h_{QD}$ = 7.7 nm (QD1) and $h_{QD}$ = 6.1 nm (QD2, QD3). Therefore, simulation results for these dot sizes are discussed.

Examples of the simulated electron and hole probability densities are plotted in Figure 2b for a lateral electric field (along x-direction) of *F* = 30 kV/cm. As a direct effect of the electric field, the electron and hole probability densities are shifted from the QD center toward different directions along the *x*-axis. The shift is stronger for the hole, which is related to the fact that electrons with smaller effective mass have a high probability density inside the barrier material and a field causes a deformation instead of a shift, rather. As a consequence, the field-induced shift is sensitive to the effective mass and the shape of a QD in a complex fashion. Furthermore, the shift is stronger for the QD with a larger extension in the x-direction. Clearly visible is the field-induced transformation of the initially localized exciton into a lateral dipole with a significant charge-carrier polarization.

**Figure 3:**
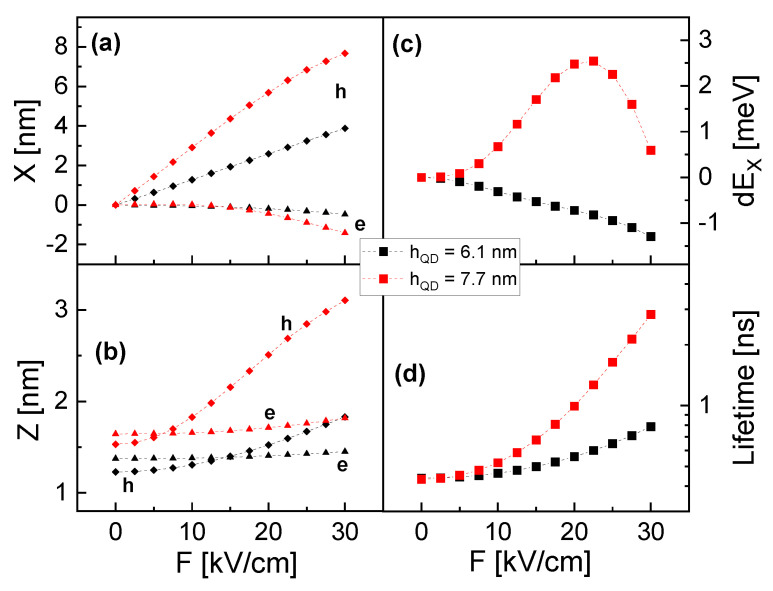
Simulation results on cone-shell QDs with different $h_{QD}$ at varied lateral electric field *F*. (**a**) Lateral position x of the electron (e) and hole (h) probability-density barycenters relative to the QD symmetry axis. (**b**) Vertical position z of the electron and hole probability-density barycenters relative to the position of $h_{QD}/2$. (**c**) Exciton Stark-shift $dE_{X}=E_{X}\left(F\right)-E_{X}\left(0\right)$. (**d**) Radiative lifetime.

The position of the electron and hole probability-density barycenters is plotted in Figure 3a,b as a function of *F*. The data already indicate at zero *F* a charge-carrier polarization in the z-direction, a shift of the charge carriers by a lateral *F* not only in x- but also along the z-direction, and a stronger *F*-induced shift for the hole in comparison to the electron (already discussed above). The first two effects are caused by the cone-shell shape of the studied QDs.

The simulated exciton energy in Figure 3c indicates a substantial influence of $h_{QD}$. $E_{X}$ decreases almost linearly with increasing *F* for the smaller CSQD, whereas for the larger dot, there is first an increase of $E_{X}$ up to *F* = 23 kV/cm, followed by a decrease. For both sizes, the *F*-dependence deviates from the often-observed parabolic Stark-shift [5,8]. For the larger CSQD, the lateral distance in *F*-direction between the electron and hole probability-density barycenters shows a continuous increase (Figure 3a), which cannot explain the increasing $E_{X}$ and the inversion of the $E_{X}\left(F\right)$ slope at *F* = 23 kV/cm. This indicates that *F* also modifies the shape of the probability densities, which determines the $E_{X}\left(F\right)$ curve up to *F* = 23 kV/cm. At higher *U*, the now decreasing $E_{X}\left(F\right)$ indicates that the charge-carrier separation becomes the dominant contribution. For the smaller CSQD, the charge-carrier deformation is less pronounced and transforms the usually parabolic decrease of $E_{X}\left(F\right)$ into an almost linear one.

The radiative (bright) lifetime $\tau_{B}$ is calculated in the limit of strong confinement from the overlap integral of the electron and hole wave functions according to Fermi’s golden rule [39]. The simulated $\tau_{B}\left(F\right)$ in Figure 3d shows a clear increase with increasing *F*, which is related to a reduction in the wave-function overlap due to an increasing charge-carrier polarization.

## 4. Experimental Results

We have investigated, in total, 12 different CSQDs from the *d* = 6 µm and the *d* = 8 µm gate structures (Figure 1d). In general, CSQDs inside the same gate structure show PL data with similar *U*-dependent trends. Data from three typical CSQDs are discussed in the following, where QD1 and QD2 are located inside the *d* = 6 µm gate structure with electric field orientation along [1-10]-direction and QD3 inside the *d* = 8 µm gate structure with field along [110].

**Figure 4:**
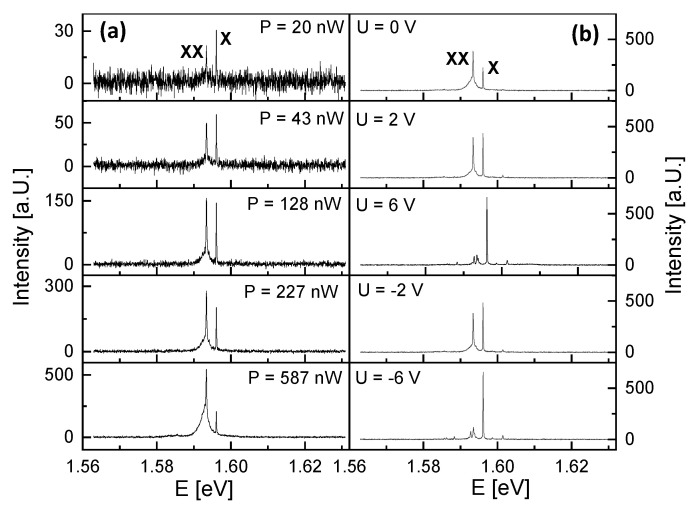
PL spectra of QD1. (**a**) *U* = 0 V and varied excitation power *P* as indicated. (**b**) *P* = 363 nW and varied lateral gate voltage *U*. The exciton (X) and biexciton (XX) peaks are marked.

Figure 4 shows PL spectra from QD1 at varied excitation power *P* and lateral gate voltage *U*. For *U* = 0 V and *P* = 20 nW, the PL spectrum in Figure 4a shows two peaks, one at *E* = 1.5968 eV and a second at 2.6 meV lower energy. An increase of *P* yields an increasing intensity of both peaks; however, the intensity of the peak at lower energy increases in strength and becomes dominant. This is a typical behavior for the exciton (X) and biexciton (XX) emission [18] and allows an identification of the peaks. Accordingly, the exciton energy is $E_{X}$ = 1.5968 eV and the X-XX splitting is 2.6 meV. This value of $E_{X}$ agrees in the simulations with a QD height of $h_{QD}$ = 7.7 nm (Figure 2a). We measure now the gate voltage dependence (Figure 4b), which demonstrates an interesting effect of the biexciton. At low absolute voltages the intensity is strong, whereas the biexciton peak is almost suppressed for *U* = ±6 V. On the other side, the exciton intensity increases with *U*. Furthermore, we note that the spectra for equal absolute values of *U* are quite similar.

**Figure 5:**
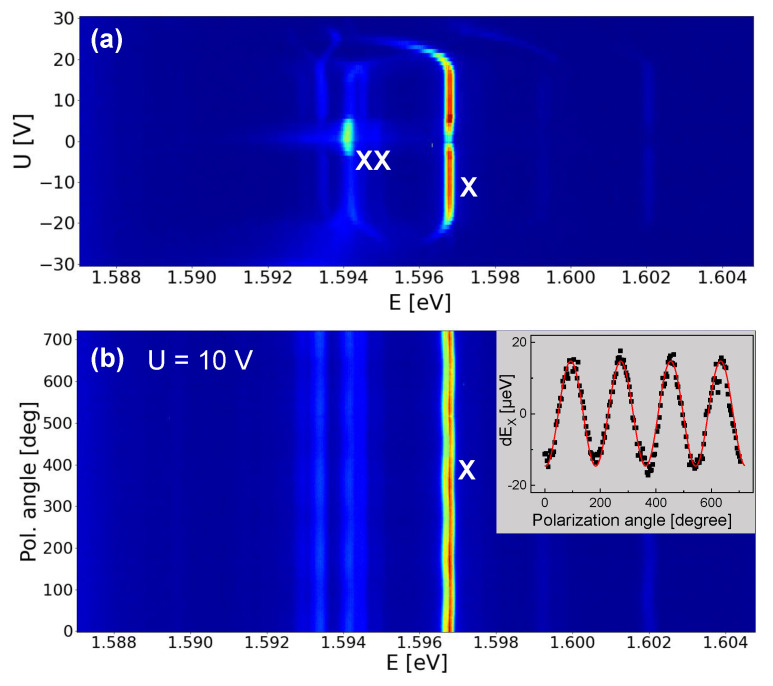
Color-coded PL spectra of QD1 at *P* = 227 nW. (**a**) The gate voltage *U* is varied without polarization filter. The exciton (X) and biexciton (XX) lines are labeled. (**b**) The polarization angle α is varied at *U* = 10 V. The inset shows the exciton energy $dE_{X}\left(\alpha \right)=E_{X}\left(\alpha \right)-\bar{E_{X}\left(\alpha \right)}$ determined using Lorentzian fits. The values of $dE_{X}$ are fitted by a sine function (red line). The exciton fine-structure splitting (FSS) of 31.4 µeV is two times the amplitude of the sine.

To illustrate the *U*-dependence over a wider range, in Figure 5a, color-coded PL spectra are plotted. The identification of the X and XX lines is addressed above. There are several additional lines, but at a low intensity. The *U*-dependent data are mostly symmetric around *U* = 0. A slight asymmetry can be caused by non-equal Schottky contacts of the lateral gate electrodes and a QD position not at the center of the gate structure. Figure 5b gives an example of a series of spectra with respective selected polarization angle α. Clearly visible is the oscillation of $E_{X}$ at varied α. This phenomenon is well known as exciton fine-structure splitting (FSS) [23,24,25,26,34]. The FSS splits the exciton peak into two orthogonally polarized components [31,32]. Since the two split peaks have a very small energy separation below the spectral resolution of our spectrometer, we measure only one peak whose center is shifted by a varied polarization angle. For a quantitative analysis of the data, we use Lorentzian fits to determine $E_{X}\left(\alpha \right)$ and fit the resulting $dE_{X}\left(\alpha \right)=E_{X}\left(\alpha \right)-\bar{E_{X}\left(\alpha \right)}$ by a sine function, with the mean energy $\bar{E_{X}\left(\alpha \right)}$ (inset in Figure 5b). The value of the FSS is two times the amplitude of the sine.

**Figure 6:**
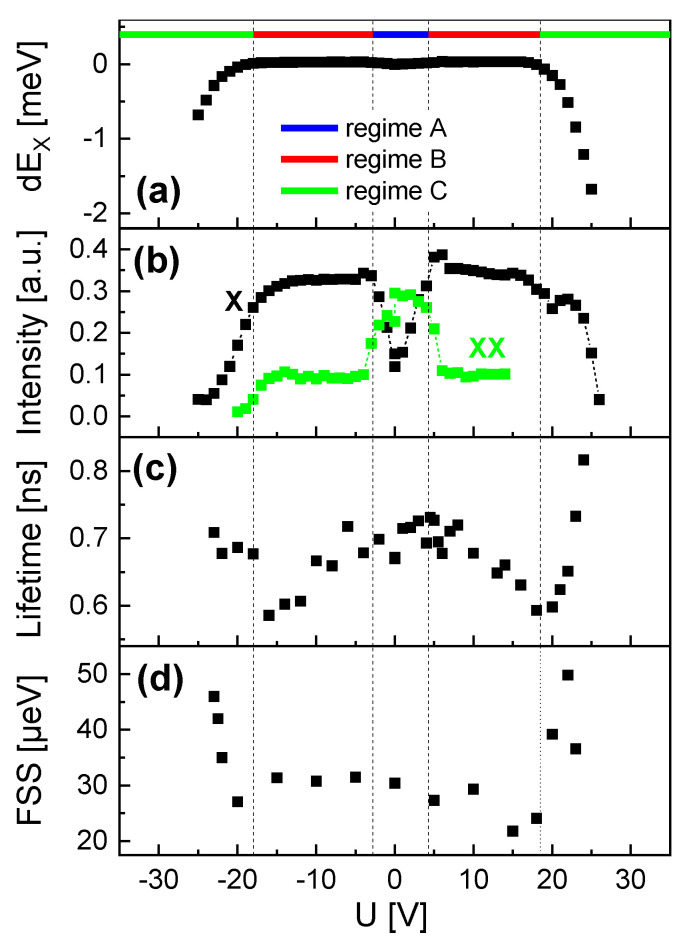
Gate voltage -dependent properties of QD1 with $E_{X}\left(U=0\right)$ = 1.5968 eV. (**a**) Stark-shift $dE_{X}=E_{X}\left(U\right)-E_{X}\left(0\right)$ of the exciton peak energy with indicated regimes. (**b**) Intensity of the exciton (X) and biexciton (XX) as the peak area determined by Lorentzian fits. (**c**) Exciton radiative lifetime. (**d**) Exciton fine-structure splitting (FSS).

The exciton peaks are quantitatively analyzed using Lorentzian fits and the resulting energy and intensity are plotted in Figure 6a,b. The data show complex *U*-dependent trends, where three regimes can be distinguished (Figure 6a). At low absolute voltages (regime A, −2.8 V ≤U≤ +4.3 V), the exciton energy $E_{X}$ increases slightly with *U* and the exciton intensity shows a clear drop. For higher absolute *U*, both $E_{X}$ and the intensity are almost constant (regime B, −18 V ≤U<−2.8 V and +4.3 V <U≤ +18.5 V). For even higher absolute voltages (regime C, U<−18 V and U> +18.5 V) $E_{X}$ and the intensity start to decrease strongly. Interestingly, the intensity of the biexciton shows in regimes A and B an inverse trend compared to the exciton and almost vanishes in regimes B and C.

In the next step, we switched to a pulsed laser mode and used an APD as a single-photon detector to measure the time dependence of the exciton peak intensity. In agreement with previous results [37], the time-dependent decay of the exciton intensity I(t) can be well fitted by biexponential decay, as follows:

(1) $$I\left(t\right)=A_{F}\exp\left(-t/\tau_{F}\right)+A_{S}\exp\left(-t/\tau_{S}\right)+I_{0}$$

with constants $A_{F}$, $A_{S}$, $I_{0}$, and the fast $\tau_{F}$ and slow $\tau_{S}$ decay times. According to Ref. [37], this allows the calculation of the radiative (bright) recombination rate $R_{B}$, as follows:

(2) $$R_{B}=+\frac{A_{F}}{A_{F}+A_{S}}\tau_{F}^{-1}+\frac{A_{S}}{A_{F}+A_{S}}\tau_{S}^{-1}$$

The corresponding radiative lifetime is $\tau_{B}=1/R_{B}$. The lifetime data from QD1 (Figure 6c) show a “W”-like *U*-dependence, with $\tau_{B}$ of about 700 ps in the center (regime A). With increasing absolute *U* in regime B, the lifetime decreases down to about 600 ps. Even higher *U* in regime C yields a strong increase of $\tau_{B}$.

The *U*-dependent FSS for QD1 is plotted in Figure 6d and shows a slight decrease with increasing absolute *U* in regime B and an almost abrupt increase in regime C.

**Figure 7:**
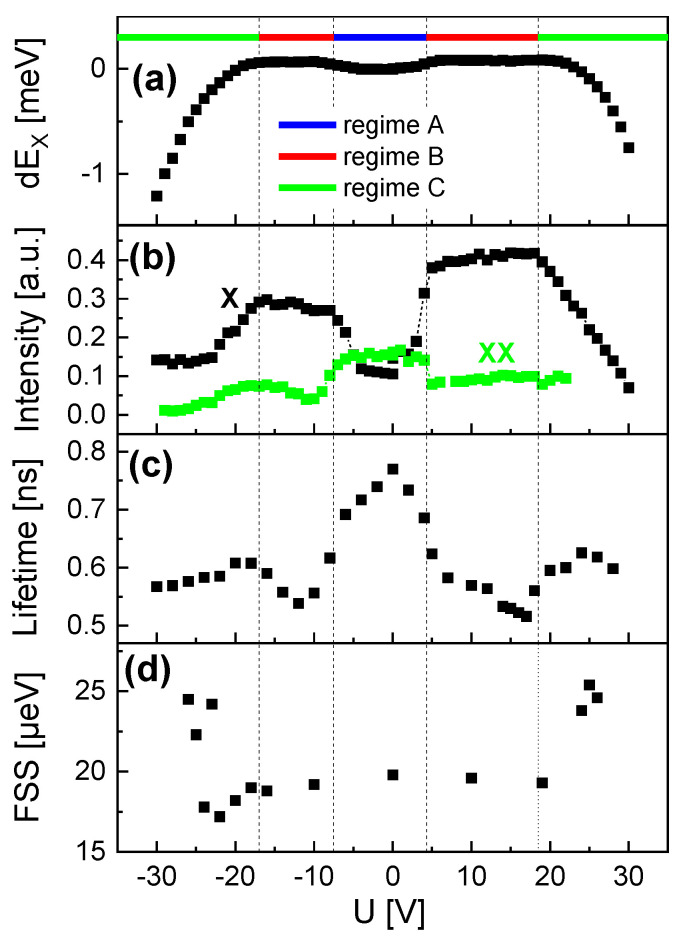
Gate voltage -dependent properties of QD2 with $E_{X}\left(U=0\right)$ = 1.6397 eV. (**a**) Stark-shift of the exciton peak energy with indicated regimes. (**b**) Intensity of the exciton (X) and biexciton (XX) peaks. (**c**) Exciton radiative lifetime. (**d**) Exciton fine-structure splitting (FSS).

Figure 7 demonstrates similar behavior for a second QD (QD2), with $E_{X}\left(U=0\right)$ = 1.6397 eV and an X-XX splitting of 3.3 meV. The value of $E_{X}$ corresponds in the simulations to a QD height of $h_{QD}$ = 6.1 nm. Again, we can distinguish three regimes. However, compared to QD1, the borders are slightly different with *U* = −7.5 V, +4.3 V for the border between regimes A and B, and *U* = −17 V, +18.5 V between regimes B and C. This deviation can be explained by a different position of the QD between the gate electrodes. In regime A, the exciton energy reduction is stronger compared to QD1 and the biexciton intensity is less pronounced. The *U*-dependent lifetime reduction from $\tau_{B}$ = 780 ps to 530 ps in regimes A and B is stronger, and regime C shows no clear increase of $\tau_{B}$. Furthermore, the FSS is slightly lower for QD2 compared to QD1 with a larger size. This effect that larger QDs have a larger FSS is known [26]. Very small FSS below 5 µeV were demonstrated on similar QDs [26,40], but those have an even smaller size. Here, a sample with larger QDs is selected, which allows a wide tunability of the charge-carrier wave functions.

**Figure 8:**
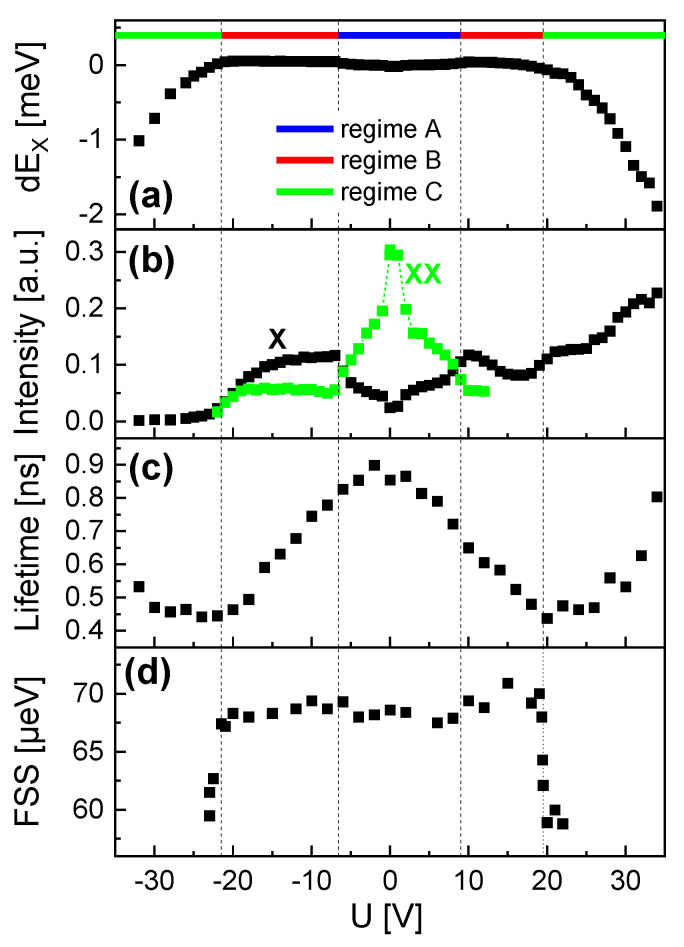
Gate voltage -dependent properties of QD3 with $E_{X}\left(U=0\right)$ = 1.6396 eV. (**a**) Stark-shift of the exciton peak energy with indicated regimes. (**b**) Intensity of the exciton (X) and biexciton (XX) peaks. (**c**) Exciton radiative lifetime. (**d**) Exciton fine-structure splitting (FSS).

For QD3, the direction of the lateral electric field is rotated by 90° in comparison to QD1 and QD2. The exciton energy is $E_{X}\left(U=0\right)$ = 1.6397 eV and the X-XX splitting 3.3 meV. This suggests a size similar to QD2. The PL data are plotted in Figure 8 and again three regimes are visible. The trends of the exciton energy and intensity as well as of the lifetime are similar compared to QD2. Noticeable deviations are the biexciton intensity which is more pronounced in regime A, and an increasing exciton intensity for positive *U* in regime C. However, the most important point is the inverse trend of the FSS in regime C. In contrast to QD1 and QD2 with an increasing FSS, the FSS of QD3 shows a clear reduction. This substantial deviation will be linked in Section 5 to the different in-plane orientations of the electric field. We note that QD3 shows a higher intrinsic FSS at *U* = 0 compared to QD1 and QD2. This is probably caused by a stronger shape anisotropy of this dot. Nevertheless, this dot was selected here since the larger FSS allows a more precise fitting. We note that other QDs in the *d* = 8 µm gate structure show a smaller intrinsic FSS like QD1 and QD2, but all of them show the reduction in regime C.

## 5. Discussion and Conclusions

We study the optical emission of exciton states in cone-shell QDs and here in detail the energy, intensity, radiative lifetime, and fine-structure splitting as a function of a lateral gate voltage. The applied gate voltage *U* induces a lateral electric field *F* that modifies the charge-carrier probability densities. In the experiments, the relation between *F* and *U* is probably nonlinear. In a most simple parallel-plate capacitor model, the field can be estimated as F=U/d, with the distance *d* = 6 µm or *d* = 8 µm between the lateral gate electrodes. However, the two metallic gates form Schottky contacts with charges in the depletion zones and at the interfaces to the semiconductor. These charges can add further electric fields or they can cause a screening of the external field induced by the gate voltage. Furthermore, a possible non-uniformity of *d* can cause a slight position-dependence of *F*. Therefore, the experimental and the simulated results are not directly comparable and we consider the simulation results for an evaluation of the more general mechanisms.

The gate voltage-driven electric field yields a polarization of the charge carriers. The polarization is mainly given by a distance *p* between the barycenters of the electron and hole probability densities. An additional contribution is caused by a deformation of the probability densities.

This charge-carrier polarization originates in the well-known Stark-shift of the exciton energy $E_{X}$, where often a parabolic field dependence is observed [5,8]. In agreement with that, an earlier study on InAs QDs in a lateral electric field demonstrates a parabolic Stark-shift [35]. However, in the present study, the clearly non-parabolic Stark-shift of the GaAs CSQDs represents an interesting finding and is attributed to the shape of the CSQDs. In a simple approximation, the electron and hole probability densities are taken as point charges at a distance *p*. At zero electric field *F* we have $p\left(F=0\right)=p_{0}$. An electric field *F* causes a charge-carrier separation and, thus, a change of *p*. Assuming a linear dependence $p\left(F\right)=p_{0}+\beta F$, we get a parabolic energy shift $dE\left(F\right)=E\left(F\right)-E\left(F=0\right)=-pF+p_{0}F=-\beta F^{2}$, with the polarizability β. For the present CSQDs, we assume two main deviations from this point-charge approximation. First, a nonlinear relation F(U) (see above), and, second, a nonlinear p(F) induced by a charge carrier shift in the lateral and vertical direction as well as by a deformation of the electron and hole probability densities.

A better model for an evaluation of the Stark-shift is the finite-element simulation that considers the shape of the CSQDs. An important outcome of the simulations is that a lateral electric field shifts the charge carriers not only in lateral but also vertically (Figure 3a,b). This finding motivates a general difference between the Stark-shift and the radiative lifetime. Both quantities depend on the charge-carrier polarization, the Stark-shift directly, and the lifetime via the overlap integral of the electron and hole wave functions according to Fermi’s golden rule [39]. However, as a major difference, the Stark-shift gauges only the lateral polarization in field direction, whereas the lifetime depends on the lateral and vertical polarization. This difference causes different *U*-dependent trends, which is clearly visible in the experimental data.

The simulations suggests for $h_{QD}$ = 7.7 nm that the trend of the $E_{X}\left(F\right)$ curve for a low *F* is controlled by a deformation of the probability densities (see Section 3). Transferring this result to the experimental *U*-dependence, we assume that the slight increase of $E_{X}$ in regime A is caused mainly by a deformation of the charge carriers and that the strong decrease in regime C is originated by an increasing lateral *p*. The almost constant $E_{X}$ in regime B is more complex and will be addressed in the following.

The inverse *U*-dependent trend of the biexciton intensity and its suppression in regimes B and C represents an interesting experimental finding. Possible explanations would be a reduced population of the biexciton state or an elongated radiative lifetime. Since the effect is also visible at higher excitation power, we assume that it is not caused by the population. Furthermore, the abrupt change in the biexcition intensity at a certain *U* is not compatible with the continuous charge-carrier deformation as is assumed above for the exciton. So, we assume for the biexcition an abrupt *U*-induced deformation of the probability density of one charge-carrier type. This yields a situation similar to the optical selection rules, where optical transitions, e.g., between s-like and p-like states, have a low probability. To better clarify this point, a more advanced simulation model which also includes biexciton states would be desirable.

The radiative (bright) lifetime $\tau_{B}$ is related to the exciton peak intensity $I=cn/\tau_{B}$, assuming a simple approximation for a low exciton generation rate *G*, with a constant *c* and the population $dn/dt=\left(1-n\right)G-n/\tau_{B}$ of the exciton state. In equilibrium (t→∞), we get dn/dt=0 and, thus, $I=c/(\tau_{B}+1/G)$. This approximation gives an explanation for the experimental trend where *I* increases with *U* in regime A whereas $\tau_{B}$ decreases. However, a closer inspection of the experimental data indicates that the voltage range for the decrease of $\tau_{B}$ is much broader than that for the increase of *I*. As a further open point, a decreasing $\tau_{B}$ is not found in the simulation results (Figure 3d).

A possible explanation for these discrepancies might be an additional vertical electric field caused by surface charges. Such a field would result in a significant vertical electron-hole polarization already at zero *U*. The simulations indicate that a lateral gate voltage induces a lateral and also a vertical shift of the charge carriers (Figure 3b), which can compensate the surface-induced polarization. This can explain why $\tau_{B}$ decreases in regime B whereas $E_{X}$ is almost constant. In detail, an increasing *U* reduces mainly the vertical charge-carrier polarization for a shorter $\tau_{B}$, whereas the lateral polarization and, thus, $E_{X}$ stays constant. In regime C, a strong reduction of $E_{X}$ is accompanied by an increasing $\tau_{B}$. This indicates a substantial *U*-induced increase, now also of the lateral polarization.

The fine-structure splitting is caused by the in-plane anisotropy of a QD, which yields a splitting of the bright exciton into two orthogonally polarized components with slightly different energies [31,32]. Since the present strain-free GaAs QDs have no strain-induced piezoelectric fields and the effect of the atomistic symmetry anisotropy is rather small [29,30], the in-plane QD shape anisotropy [26] is suggested as the major source for the intrinsic FSS at *U* = 0. A previous study [36] supports this and indicates that the CSQDs are slightly anisotropic with the radius $r_{QD}$ along the [1-10]-direction, being about 1.2 times longer than along [110]. At $\left|U\right|>0$, the electric field-induced formation of a dipole and the corresponding charge-carrier polarization yields an additional contribution to the FSS. Here, the only slowly varying FSS in regimes A and B supports the assumption of an only negligible in-plane polarization in these regimes. In regime C, the enhanced FSS of QD1 and QD2 is attributed to a field-induced polarization in the direction of the CSQD elongation, which enhances the anisotropy. On the other side, the reduction in the FSS for QD3 indicates a polarization perpendicular to the direction of the CSQD elongation and a corresponding reduction in the anisotropy.

In summary, simulations predict for the studied GaAs CSQDs that the charge carriers form a lateral dipole in an external lateral electric field. In an experimental evaluation, the exciton Stark-shift, radiative lifetime, and FSS are studied as function of a lateral gate voltage *U*. The experimental data show complex trends with three *U*-dependent regimes. These are assumed to be dominated by charge-carrier deformation at low *U*, a vertical charge-carrier polarization at medium *U*, and a lateral charge-carrier polarization at high *U*. This lateral polarization forms a lateral dipole, which can either enhance or compensate the intrinsic FSS induced by the QD shape anisotropy, dependent on the in-plane orientation of the electric field. Furthermore, an additional electric field induced by surface charges is assumed and the suppression of the biexcition peak by a lateral gate voltage is observed. 

## Data Availability

The data presented in this study are available on request from the corresponding author.
